# Community composition and functional prediction of prokaryotes associated with sympatric sponge species of southwestern Atlantic coast

**DOI:** 10.1038/s41598-021-88288-3

**Published:** 2021-05-05

**Authors:** C. C. P. Hardoim, A. C. M. Ramaglia, G. Lôbo-Hajdu, M. R. Custódio

**Affiliations:** 1 Institute of Biosciences, São Paulo State University, Coastal Campus of São Vicente, São Paulo, Brazil; 2 Department of Genetic, Biology Institute Roberto Alcântara Gomes, Rio de Janeiro State University, Rio de Janeiro, Brazil; 3 Department of Physiology, Center for Marine Biology, Biosciences Institute and NP-Biomar, São Paulo University, São Paulo, Brazil

**Keywords:** Microbial ecology, Microbiome, Symbiosis

## Abstract

Prokaryotes contribute to the health of marine sponges. However, there is lack of data on the assembly rules of sponge-associated prokaryotic communities, especially for those inhabiting biodiversity hotspots, such as ecoregions between tropical and warm temperate southwestern Atlantic waters. The sympatric species *Aplysina caissara*, *Axinella corrugata*, and *Dragmacidon reticulatum* were collected along with environmental samples from the north coast of São Paulo (Brazil). Overall, 64 prokaryotic phyla were detected; 51 were associated with sponge species, and the dominant were Proteobacteria, Bacteria (unclassified), Cyanobacteria, Crenarchaeota, and Chloroflexi. Around 64% and 89% of the unclassified operational taxonomical units (OTUs) associated with Brazilian sponge species showed a sequence similarity below 97%, with sequences in the Silva and NCBI Type Strain databases, respectively, indicating the presence of a large number of unidentified taxa. The prokaryotic communities were species-specific, ranging 56%–80% of the OTUs and distinct from the environmental samples. Fifty-four lineages were responsible for the differences detected among the categories. Functional prediction demonstrated that *Ap. caissara* was enriched for energy metabolism and biosynthesis of secondary metabolites, whereas *D. reticulatum* was enhanced for metabolism of terpenoids and polyketides, as well as xenobiotics’ biodegradation and metabolism. This survey revealed a high level of novelty associated with Brazilian sponge species and that distinct members responsible from the differences among Brazilian sponge species could be correlated to the predicted functions.

## Introduction

The phylum Porifera consists of sessile and filter-feeding animal communities, which are among the oldest living Metazoans, dating back to approximately 700 Myr ago^1^. The class Demospongiae encompasses 85% of the 9345 valid sponge species described thus far^2^. One of the challenges of in studying these animals is that several species lack morphological features that assist classical taxonomy. For instance, the families Aplysinidae and Axinellidae are notoriously difficult to identify due to the lack of diagnostic morphological features^3–6^. Therefore, DNA barcoding has been used to aid species identification^7^. Because of their intrinsic characteristics, sponges are known to perform several services contributing to the functioning and health of benthic ecosystems^8,9^. Marine sponges also harbour diverse and complex prokaryotic communities^9,10^, and in certain cases, up to 38% of the sponge wet weight is composed of bacterial cells^11^. The most comprehensive study performed so far detected between 41 and 72 recognised and candidate prokaryotic phyla associated with 269 sponge species encountered worldwide, but not from the Brazilian coast^10,12^.

The field of sponge microbiology in Brazil is still underdeveloped. The Brazilian marine coast encompasses approximately 7367 km and currently has 597 sponge species distributed within the four classes, which correspond to approximately 6% of the global sponge diversity^13^. Among them, 113 sponge species are endemic to the Brazilian coast^13^. The number of registers in this region is constantly increasing due to new records and species^14–16^. However, only four prior studies have assessed the prokaryotic communities associated with the Brazilian marine sponges, which were performed with species from the coast of Rio de Janeiro using less robust techniques^17–20^.

To assess the importance of deterministic components for prokaryotic community assembly in southwestern Atlantic, three common sponge species from São Paulo state were investigated. This coastal area has approximately 600 km of extension, divided into littoral south and north, and encompasses the transition between tropical and warm temperate southwestern Atlantic marine ecoregions^21^. This region is considered one of the most significant benthic biodiversity hotspots and ecosystem services globally but is relatively unknown. It is characterised by microtides, a transition between low to high productivity, where sponges are one of the most important eco-engineers. However, anthropogenic threats, such as urbanisation, invasive species, climate change, and mining, are affecting the three-dimensional living structures offered by sessile animals^22^. The city of São Sebastião is located on the north coast and hosts over 70 sponge species described, belonging to the classes Calcarea and Demosponges^23^, and might be considered a local hotspot of sponge diversity in Brazil. The sympatric sponges *Aplysina caissara* (Pinheiro & Hajdu, 2001), *Dragmacidon reticulatum* (Ridley & Dendy, 1886), and *Axinella corrugata* (George & Wilson, 1919), along with the environmental samples (seawater and sediment) were collected from this region. *Aplysina caissara* has a narrow distribution and is endemic to southern and southeastern Brazil^4^. Even though a sponge with similar characteristics as *Ap. caissara* was discovered in the Guyana shelf, certain features made the specimen identification difficult^24^; hence, we considered its original distribution. *Ax. corrugata* and *D. reticulatum* are widely distributed along the Brazilian coast and the Caribbean Sea^2^. The main questions in this study were (***i***) whether DNA barcoding was capable of separating the sponge species, (***ii***) whether the prokaryotic communities associated with the sponge species can be distinguished from the ones detected in the environmental samples, (***iii***) whether the sponge species exhibited host specificity, (***iv***) which prokaryotic lineages are responsible for the variability detected in the categories, and (***v***) which predicted functional patterns are enriched in each category.

## Results

### Sponge barcoding

**Figure 1:**
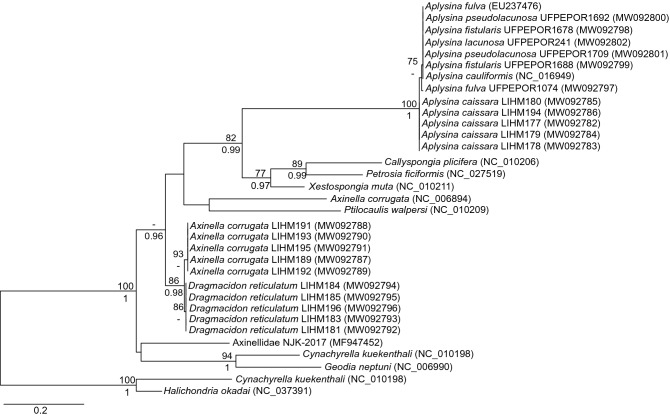
Phylogenetic inference of the Axinellidae and Aplysinidae families based on the cytochrome b. The Maximum Likelihood tree (-ln likelihood: -2655.123092) is shown. ML bootstrap values (> 75%) and Bayesian posterior probabilities (> 0.95) are shown above and below branches, respectively.

Analysis of 364 bp-long sequences of the mitochondrial cytochrome b (cob) obtained from all 15 specimens showed no intraspecific variations among our sequences of *Ap. caissara*, *Ax. corrugata,* and *D. reticulatum*. A genetic distance (*p*-distance) of 0.82% was observed among *Ap. caissara* and other *Aplysina* species, whereas 18.13% was detected between *Ax. corrugata* from GenBank and our sequences. For *Ap. caissara* and *D. reticulatum*, these were the first cob sequences reported and, as such, no prior sequence of this gene was available from the National Center for Biotechnology Information (NCBI) for comparison. Phylogenetic reconstructions based on Maximum Likelihood and Bayesian inferences indicated that each sponge species formed a robust cluster (Fig. 1), suggesting that the cob gene is efficient in differentiating *Ap. caissara* from other *Aplysina* species.

### Analysis of the 16S rRNA gene

A total of 3,814,873 V4-region of the 16S rRNA gene sequences were obtained on an Illumina MiSeq platform. After denoising, quality filtering, and removal of chimera and undesirables, a total of 2,950,757 16S rRNA sequences were further analysed with Mothur v.1.44. Then, singletons were also removed from the dataset resulting in 2,865,820 sequences that were further rarefied to the same library depth of 70,314 sequences, resulting in 1,757,850 sequences (Supplementary Table S2). These were assigned to 43,947 OTUs at 97% sequence similarity.

### Prokaryotic alpha diversity

The rarefaction curve demonstrated that except for *Ap. caissara* and sediment, all the other categories reached a plateau with the sequence depth used (Supplementary Fig. S1). Sediment presented the highest observed CHAO richness, followed by seawater, *Ap. caissara*, *Ax. corrugata,* and *D. reticulatum* (Table 1). Analysis of variance (ANOVA) revealed that the categories differed from each other, and this difference was detected between each sponge species and sediment as well as seawater and sediment (*p* < 0.001), (Supplementary Table S3). Sediment also showed the highest estimated CHAO richness, followed by seawater, *Ap. caissara*, *Ax. corrugata*, and *D. reticulatum*. These categories were significantly different according to ANOVA analysis, and it was detected between the categories sediment and each sponge species, seawater and *Ax. corrugata*, seawater and *D. reticulatum*, and seawater and sediment (*p* < 0.001), *Ap. caissara* and *D. reticulatum*, and seawater and *Ap. caissara* (*p* < 0.01) (Supplementary Table S3). For the ACE index, the highest richness was observed in sediment, followed by seawater, *Ap. caissara*, *Ax. corrugata*, and *D. reticulatum* (Table 1). These categories differed significantly according to ANOVA. This dissimilarity was registered between sediment and each sponge species, seawater and *Ax. corrugata*, seawater and *D. reticulatum*, and seawater and sediment (*p* < 0.001), *Ap. caissara* and *Ax. corrugata*, *Ap. caissara* and *D. reticulatum*, and *Ap. caissara* and seawater (*p* < 0.01) (Supplementary Table S3).

**Table 1:** Values for richness, diversity and evenness indices.

Samples (index ± standard deviation (SD))	Ac	Ax	Dr	SW	SD
Sobs ± SD	2763.2 ± 101.11	2331.8 ± 224.69	2312.8 ± 619.24	2848.4 ± 76.41	10,886.6 ± 189.5
Chao ± SD	5106.27 ± 221.91	4200.5 ± 442.6	3822.98 ± 341.84	6158.08 ± 326.54	17,048.26 ± 345.12
ACE ± SD	5710.87 ± 349.0	4457.02 ± 511.94	4175.33 ± 183.33	6952.41 ± 399.28	18,513.73 ± 462.99
Shannon ± SD	4.68 ± 0.021	3.85 ± 0.332	4.95 ± 0.67	3.54 ± 0.076	7.46 ± 0.032
Inverse Simpson ± SD	0.96 ± 0.003	0.87 ± 0.037	0.96 ± 0.033	0.86 ± 0.007	0.99 ± 0.00024
Pielou´s evenness ± SD	0.60 ± 0.005	0.50 ± 0.04	0.64 ± 0.07	0.44 ± 0.008	0.80 ± 0.002

Ac: *Aplysina caissara*, Ax: *Axinella corrugata*, Dr: *Dragmacidon reticulatum*, SW: seawater, SD: sediment, Sobs: estimated richness.

Using the Shannon diversity index, the highest and lowest values were observed in sediment and seawater, respectively (Table 1). There was a significant difference among the categories according to ANOVA; this was registered between sediment and each sponge species, *D. reticulatum* and seawater, seawater and sediment (*p* < 0.001), *Ap. caissara* and *Ax. corrugata*, *Ax. corrugata* and *D. reticulatum*, and *Ap. caissara* and seawater (*p* < 0.01) (Supplementary Table S3). The sediment again showed the highest Simpson diversity index, followed by *Ap. caissara*, *D. reticulatum*, *Ax. corrugata*, and seawater (Table 1). According to ANOVA, the categories were significantly different and this was registered between *Ap. caissara* and *Ax. corrugata*, *D. reticulatum* and *Ax. corrugata*, seawater and *Ap. caissara*, seawater and *D. reticulatum*, *Ax. corrugata* and sediment, and seawater and sediment (Supplementary Table S3).

The Pielou’s evenness index was the highest for sediment and the lowest for seawater (Table 1). The samples were significantly different according to ANOVA and this was observed between the categories, sediment and each sponge species, and between *Ap. caissara* and seawater, *D. reticulatum* and seawater, *Ax. corrugata* and *D. reticulatum*, seawater and sediment (*p* < 0.001), and *Ap. caissara* and *Ax. corrugata* (*p* < 0.01) (Supplementary Table S3).

### Prokaryotic community composition

**Figure 2:**
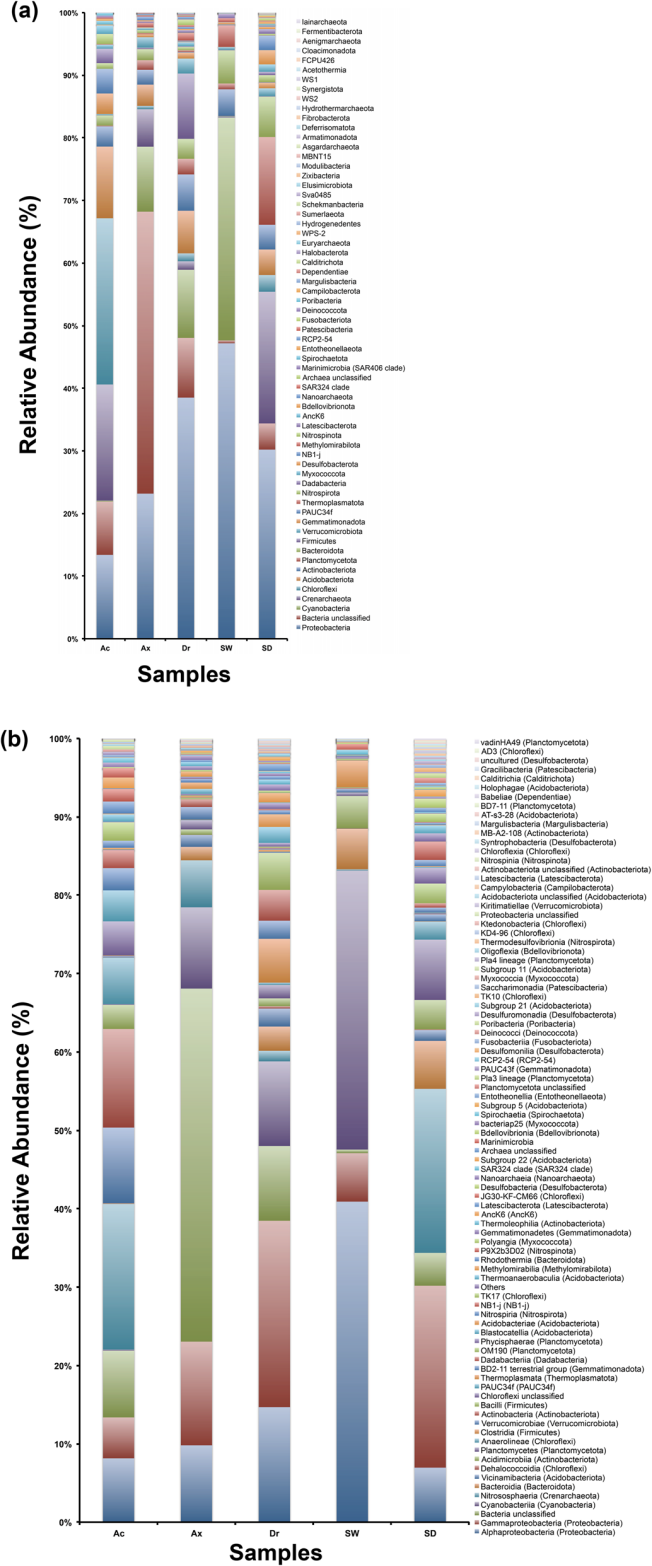
Phylum-(**a**) and class-level (**b**) prokaryotic community composition in marine sponges, seawater, and sediment. Compositional data for *Ap. caissara* (Ac), *Ax. corrugata* (Ax), *D. reticulatum* (Dr), seawater (SW), and sediment (SD) are shown. Results obtained using pooled replicate samples (n = 5) within each sample category are displayed. (**a**) All 64 prokaryotic phyla are presented. (**b**) Classes with relative abundance > 0.01% are shown, while relative abundance < 0.01% are grouped within others (88 classes).

In total, 64 prokaryotic phyla were detected among all samples (Fig. 2a, Supplementary Table S4a). Sediment samples comprised the most diverse community with 59 phyla, followed by *D. reticulatum*, seawater, *Ax. corrugata*, and *Ap. caissara* with 49, 47, 41, and 40 phyla, respectively (Supplementary Table S4a). Overall, the most abundant phyla were Proteobacteria (average relative abundance of 30.50%), Bacteria (unclassified, 13.55%), Cyanobacteria (11.48%), Crenarchaeota (9.41%), and Chloroflexi (6.21%) (Fig. 2a, Tables S4a, S5a). Chloroflexi (26.54%), Crenarchaeota (18.56%), and Proteobacteria (13.40%) were the most abundant phyla associated with *Ap. caissara* (Fig. 2a, Tables S4b, S5a). For *Ax. corrugata*, the communities were dominated by Bacteria (unclassified, 45.03%), Proteobacteria (23.18%), and Cyanobacteria (10.37%) (Fig. 2a, Tables S4c, S5a). The most abundant phyla associated with *D. reticulatum* were Proteobacteria (38.52%), Cyanobacteria (10.9%), and Firmicutes (10.42%) (Fig. 2a, Tables S4d, S5a). The most abundant phyla detected in seawater were Proteobacteria (47.2%) and Cyanobacteria (35.6%) (Fig. 2a, Tables S4e, S5a). Sediment was dominated by Proteobacteria (30.21%), Crenarchaeota (21.04%), and Planctomycetota (14%) (Fig. 2a, Tables S4f., S5a). Acidobacteriota, AncK6, Chloroflexi, Dadabacteria, Gemmatimonadota, Nitrospinota, PAUC34f, Poribacteria, and Spirochaetota were statistically (*p* < 0.05) more abundant in *Ap. caissara* when compared to all other categories. Additionally, the phyla Crenarchaeota, Deinococcota, and Entotheonellaeota were statistically (*p* < 0.05) more enhanced in *Ap. caissara* as compared specifically to *Ax. corrugata* and *D. reticulatum*. Only Bacteria (unclassified) was significantly (*p* < 0.05) abundant in *Ax. corrugata* when compared to the other sponge species. Four phyla (Actinobacteriota, Archaea (unclassified), Firmicutes, and Fusobacteriota) were significantly (*p* < 0.05) enriched in *D. reticulatum* as compared to *Ap. caissara* and *Ax. corrugata*. The phyla that were significantly (*p* < 0.05) more abundant in the axinellids species than *Ap. caissara* were Cyanobacteria, Desulfobacterota, Marinimicrobia, Methylomirabilota, Planctomycetota, Proteobacteria, RCP2-54, SAR324, Thermoplasmatota, and Verrucomicrobiota.

Overall, 169 classes of prokaryotes were retrieved (Fig. 2b, Supplementary Table S4b). The most diverse community composition was observed in the sediment with 151 classes, followed by *D. reticulatum*, seawater, *Ax. corrugata*, and *Ap. caissara* with 126, 113, 105, and 95 classes, respectively (Supplementary Table S4b). The most dominant classes were Alphaproteobacteria (16.1%), Gammaproteobacteria (14.3%), Bacteria (unclassified, 13.54%), and Cyanobacteriia (11.38%), (Fig. 2b, Supplementary Tables S4a, S5b). For *Ap. caissara*, the most abundant classes were Nitrososphaeria (18.56%) and Dehalococcoidia (12.58%) (Fig. 2b, Tables S4b, S5b). The most dominant classes associated with *Ax. corrugata* were Bacteria (unclassified, 45.03%), followed by Gammaproteobacteria (13.25%), and Cyanobacteriia (10.37%) (Fig. 2b, Tables S4c, S5b). The classes Gammaproteobacteria (23.77%), Alphaproteobacteria (14.7%), and Cyanobacteriia (10.8%) were most abundantly associated with *D. reticulatum* (Fig. 2, Tables S4d, S5b). For seawater, the dominant classes were Alphaproteobacteria (41%) and Cyanobacteriia (35.6%) (Fig. 2b, Tables S4e, S5b). The classes Gammaproteobacteria (23.24%) and Nitrososphaeria (21%) were abundantly detected in sediment (Fig. 2b, Tables S4f., S5b). Anaerolineae, Dehalococcoidia, and Vicinamibacteria were significantly (*p* < 0.05) enhanced in *Ap. caissara* compared to all other categories. These classes, along with Acidimicrobiia and Nitrososphaeria, were significantly (*p* < 0.05) abundant in *Ap. caissara* when compared to axinellids. In contrast, Actinobacteria, Bacilli, Bacteroidia, Cyanobacteria, Planctomycetes, and Verrucomicrobiae (Verrucomicrobiota) were significantly (*p* < 0.05) enhanced in axinellids when compared to *Ap. caissara*. For *Ax. corrugata*, only Bacteria (unclassified) was significantly (*p* < 0.05) abundant when compared to the other sponge species.

To obtain insights regarding the unclassified lineages associated with *Ap. caissara*, *Ax. corrugata*, and *D. reticulatum*, further analyses were performed at the operational taxonomic unit (OTU) level. Overall, from a total of 19,978 OTUs assigned to sponge species, 10,796 were unclassified at certain level of taxonomic affiliation (*i.e.* from phylum to genus). The NCBI Type Strain database revealed that 89.24% of these previously unclassified OTUs had similarity below 97%. Among them, 0.86% and 2.43% of Proteobacteria and Bacteroidota, respectively, have been detected in other sponge species (Table S6a-b). Furthermore, 40 unclassified OTUs did not fulfil our BLASTn requirements with no identified match against the NCBI Type Strain database (Table S6b). However, the Silva database indicated that 63.58% of the unclassified OTUs had similarity below 97%. Among them, 92.3, 68.2, 66.6, 58.2, and 29.96% of Crenarchaeota, Chloroflexi, Spirochaetota, Bacteria (unclassified), and Proteobacteria, respectively, have been identified in other sponge species (Table S6c-d).

### Specificities and commonalities: shared and exclusive OTUs

**Figure 3:**
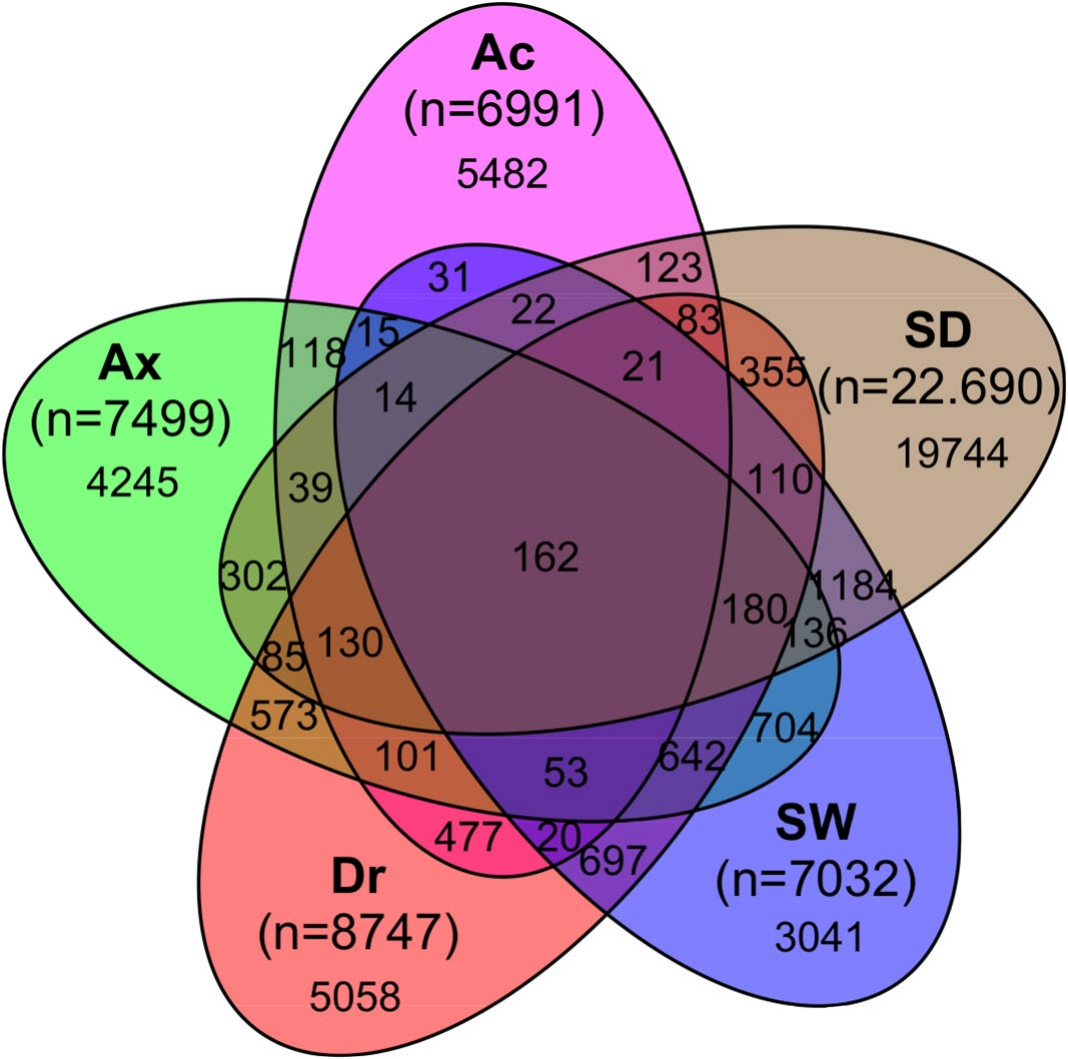
Venn diagram. All OTUs detected in *Ap. caissara* (Ac, magenta), *Ax. corrugata* (Ax, green), *D. reticulatum* (Dr, red), seawater (SW, blue), and sediment (SD, brown).

The Venn diagram revealed that the most OTUs (n = 37,570) were specific for each sample category and a few (162 OTUs) were shared among them (Fig. 3). For *Ap. caissara*, 79.55% of the communities were specific to this host species and the most abundant OTUs were affiliated to Bacteria (unclassified, 1147 OTUs in 6761 sequences), Dehalococcoidia (544 OTUs in 4413 sequences), Alphaproteobacteria (486 OTUs in 2227 sequences), and Vicinamibacteria (418 OTUs in 2055 sequences) (Supplementary Table S7a). For *Ax. corrugata*, 56.6% of the communities were specific to this species and the most dominant OTUs were affiliated to Bacteria (unclassified, 1471 OTUs in 12,979 sequences), Gammaproteobacteria (625 OTUs in 5094 sequences), and Alphaproteobacteria (404 OTUs in 6660 sequences) (Supplementary Table S7b). For *D. reticulatum*, 57.8% of the communities were specific to this host and the most dominant OTUs were affiliated to Gammaproteobacteria (1012 OTUs in 11,785 sequences), Bacteria (unclassified, 597 OTUs in 4723 sequences), Alphaproteobacteria (504 OTUs in 7422 sequences), and Clostridia (Firmicutes, 500 OTUs in 8514 sequences) (Supplementary Table S7c).

In the category of environmental samples, 43.24% of the communities were specifically detected in seawater and the dominant OTUs were affiliated to Alphaproteobacteria (1211 OTUs in 3203 sequences) and Cyanobacteriia (518 OTUs in 1156 sequences) (Supplementary Table S7d). For sediment, 87% of the communities were specific to it and the most dominant OTUs were affiliated to Gammaproteobacteria (3253 OTUs in 37,716 sequences), Bacteria (unclassified, 2407 OTUs in 12,758 sequences), Planctomycetes (1532 OTUs in 15,522 sequences), Nitrososphaeria (1263 OTUs in 11,283 sequences), Bacteroidia (1219 OTUs in 10,729 sequences), and Alphaproteobacteria (1087 OTUs in 9734 sequences) (Table S7e).

The core (composed of OTUs present in all categories, but not in all replicates) corresponded to 0.36% of the communities present in all categories (Fig. 3), and contained most sequences (42.12%, Supplementary Table S7f.). Among them, the most abundant OTUs were affiliated to Alphaproteobacteria (33 OTUs in 175,269 sequences), Gammaproteobacteria (28 OTUs in 36,947 sequences), Bacteroidia (20 OTUs in 19,305 sequences), Cyanobacteriia (10 OTUs in 188,769 sequences), and Planctomycetes (nine OTUs in 5732 sequences) (Supplementary Table S7f).

### Linear discriminant analysis effect size (LEfSe) analysis

**Figure 4:**
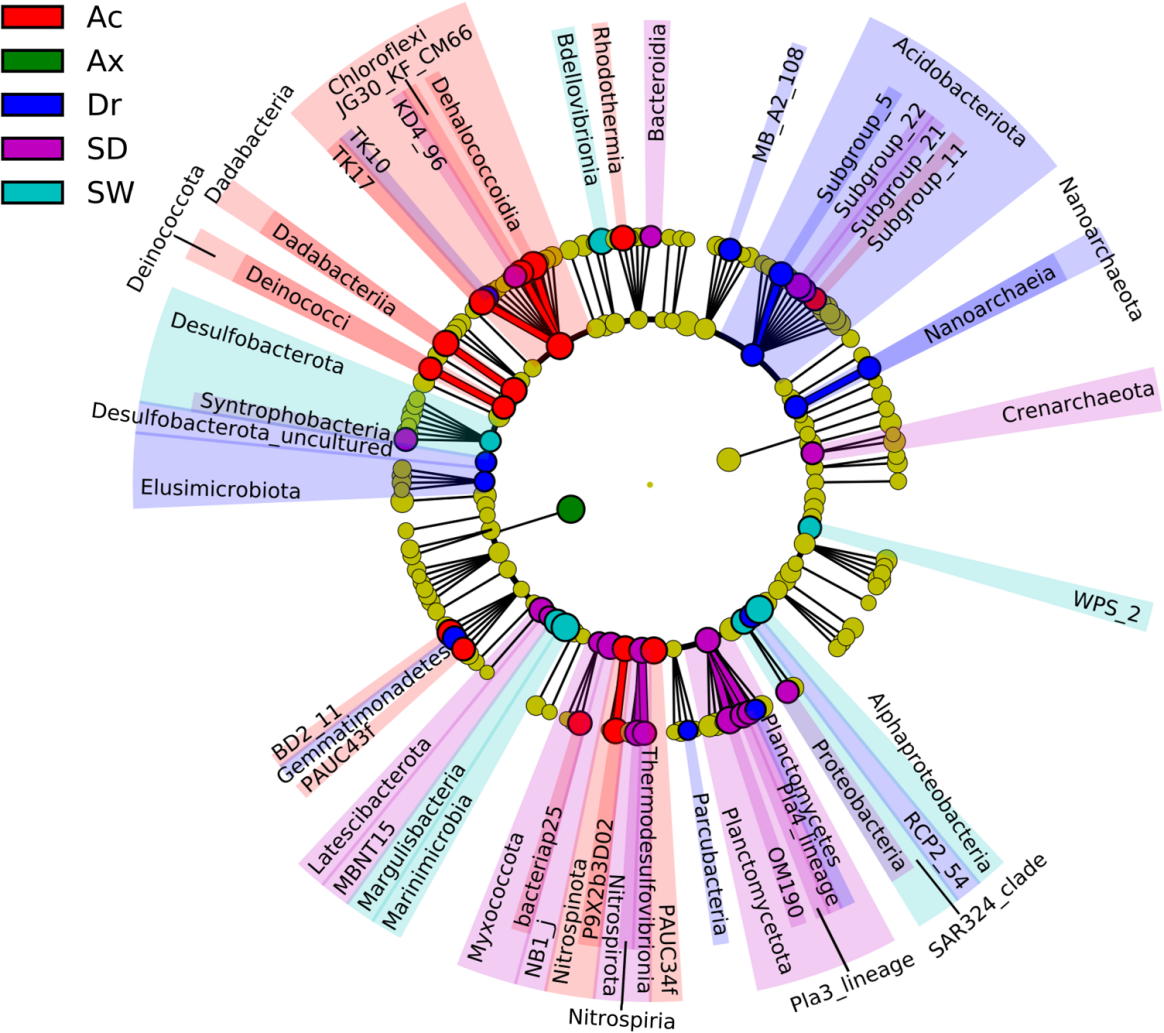
The linear discriminant analysis (LDA) effect size (LEFSe) analysis. Taxonomic representation of statistically and biologically consistent differences among *Ap. caissara* (Ac), *Ax. corrugata* (Ax), *D. reticulatum* (Dr), seawater (SW), and sediment (SD).

Fifty-four taxonomic affiliations with a phylum-class-levels categorisation had the linear discriminant analysis (LDA) score above two, which explained the variability observed in the prokaryotic communities associated with sponge species and detected in environmental samples. Sediment had 18 lineages, followed by *Ap. caissara*, *D. reticulatum*, seawater, and *Ax. corrugata* with 16, 12, seven, and one, respectively (Supplementary Table S8). The lineages are presented in the order of higher to lower LDA scores. For *Ap. caissara,* the lineages were affiliated to Dehalococcoidia, Chloroflexi, PAUC34f, BD2_11, TK17, Dadabacteriia, Dadabacteria, P9X2b3D02, Nitrospinota, Rhodothermia, JG30_KF_CM66, bacteriap25, PAUC43f, Subgroup_11, Deinococcota, and Deinococci (Fig. 4, Supplementary Table S8). In the case of *Ax. corrugata*, the only lineage with LDA score above two was Bacteria (unclassified) (Fig. 4, Supplementary Table S8). For *D. reticulatum*, lineages were affiliated to Gemmatimonadetes, Subgroup_5, RCP2_54, Elusimicrobiota, Desulfobacterota (uncultured), Planctomycetes, Parcubacteria, Nanoarchaeota, Nanoarchaeia, MBA_A2_108, Acidobacteriota, and TK10 (Fig. 4, Supplementary Table S8). In seawater, the bacterial lineages were Marinimicrobia, SAR324, Bdellovibrionia, Margulisbacteria, Proteobacteria (unclassified), WPS_2, and Desulfobacterota (Fig. 4, Supplementary Table S8). In sediment, the significant prokaryotic lineages were OM190, NB1_j, Nitrospirota, Subgroup_22, Nitrospiria, Plactomycetota, Pla3_lineage, Latescibacterota, Pla4_lineage, MBNT15, Syntrophobacteria, Thermodesulfovibrionia, Subgroup_21, KD4_96, Alphaproteobacteria, Myxococcota, Crenarchaeota, and Bacteroidia (Fig. 4, Supplementary Table S8).

### Ordination of prokaryotic OTUs

**Figure 5:**
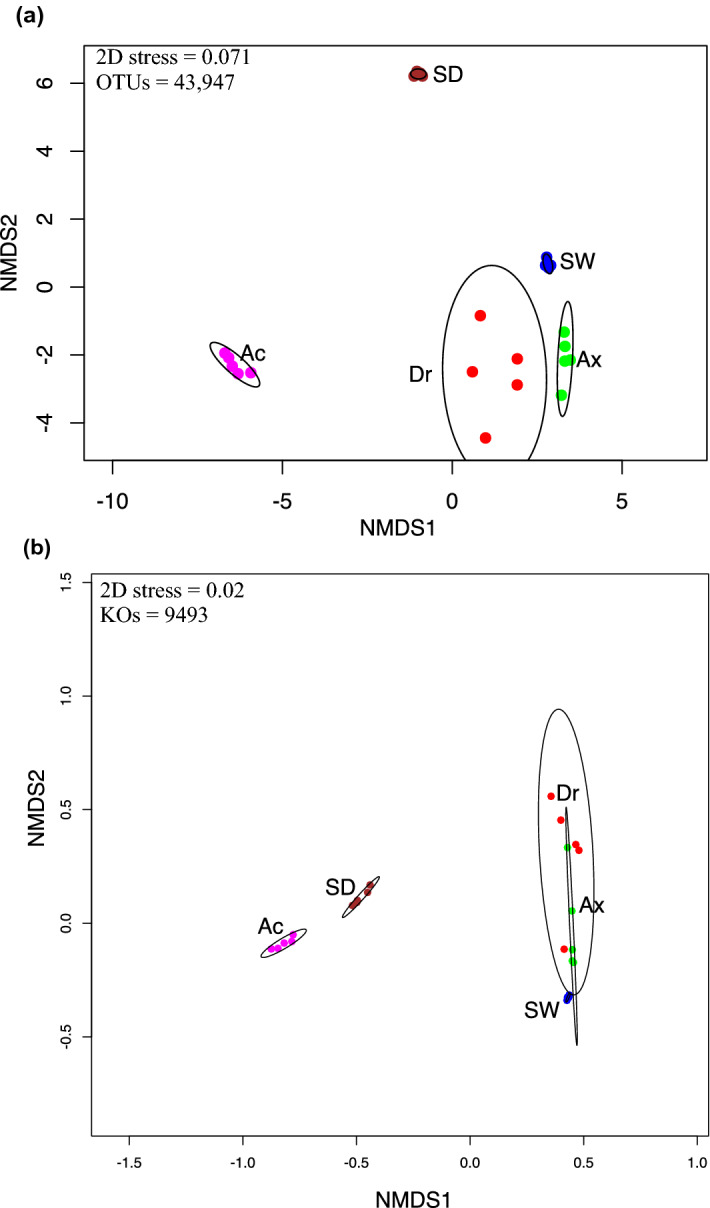
Non-metric multidimensional scaling (nMDS) plots. (a) nMDS based on Bray–Curtis distances calculated from the normalized 97% OTU table and (b) predicted KEGG Orthologs for each sponge species, seawater, and sediment. *Ap. caissara* (Ac), *Ax. corrugata* (Ax), *D. reticulatum* (Dr), seawater (SW), and sediment (SD).

The Bray–Curtis dissimilarity used in non-metric multidimensional scaling (nMDS) revealed three main groups with all replicates from: (***i***) *Ap. caissara*, (***ii***) sediment, and (***iii***) *Ax. corrugata*, *D. reticulatum*, and seawater (Fig. 5a). Replicates from *Ap. caissara*, seawater, and sediment were grouped together, whereas a more dissimilar pattern was observed among replicates from *Ax. corrugata* and *D. reticulatum* (Fig. 5a). The differences observed in Bray–Curtis dissimilarity among sponge species, seawater, and sediment was significant (*p* < 0.001) as confirmed by ADONIS.

### Functional prediction

The genomes included in this analysis encompassed one archaeal and four bacterial phyla for sponges, one archaeal and eight bacterial phyla for seawater and, three archaeal and four bacterial phyla for sediment (Supplementary Table S1b-d). In marine sponge, 25 genomes were affiliated to Streptomyces and 17 to Rhodobacteraceae (Supplementary Table S1b). In seawater, the most abundant genomes were affiliated to Vibrionaceae (n = 199), Rhodobacteraceae (n = 128), Flavobacteriaceae (n = 81), and Pseudoalteromonadaceae (n = 45) (Supplementary Table S1c). In sediment, the dominant genomes were affiliated to Flavobacteriaceae (n = 32), Rhodobacteraceae (n = 24), Vibrionaceae (n = 19), and Streptomyces (n = 12) (Supplementary Table S1d).

In total, 9493 Kyoto Encyclopedia of Genes and Genomes (KEGG) Orthologs (KOs) were predicted to be present among sponge species, seawater, and sediment (Supplementary Table S9). The nMDS constructed with the relative abundance of KOs revealed a similar pattern of distribution as observed in the 16S rRNA gene; all replicates from the categories *Ap. caissara*, seawater, and sediment were each grouped together, whereas replicates from *Ax. corrugata* and *D. reticulatum* were more dissimilar and clustered together (Fig. 5b). ADONIS revealed that the observed differences in the functional predictions among sponge species, seawater, and sediment were significant (*p* < 0.001).

In total, 392 enriched pathways were detected; according to LEfSe analysis, 253 were significantly distinct among categories. These were distributed as follows: *Ap. caissara* with 104 significantly (*p* < 0.05) distinct predicted pathways, followed by *D. reticulatum*, seawater, *Ax. corrugata*, and sediment with 60, 59, 16, and 14 distinct pathways, respectively (Supplementary Table S10). In *Ap. caissara* the most abundant predicted pathways were related to (***i***) biosynthesis of secondary metabolites (e.g. clavulanic biosynthesis, flavonoid biosynthesis, isquinoline alkaloid biosynthesis, and stilbenoid, diarylheptanoid, and gingerol biosynthesis), (***ii***) viral infectious diseases (e.g. hepatitis B and C, herpes simplex infection, and influenza A), (***iii***) xenobiotics biodegradations and metabolism (e.g. ethylbenzene degradation, naphthalene degradation, nitrotoluene degradation, polycyclic aromatic hydrocarbon degradation), (***iv***) energy metabolism pathways (i.e. carbon fixation pathways in prokaryotes, methane, nitrogen, oxidative phosphorylation, and sulphur metabolism), and (***v***) metabolism of terpenoids and polyketides (i.e. biosynthesis of ansamycins, biosynthesis of vancomycin group antibiotics, polyketide sugar unit biosynthesis, terpenoid backbone biosynthesis) (Supplementary Table S10). For *Ax. corrugata*, predicted pathways were registered in: (***i***) xenobiotics biodegradation and metabolism (i.e. chlorocyclohexane and chlorobenzene degradation, drug metabolism—cytochrome P450, drug metabolism—other enzymes, and metabolism of xenobiotics by cytochrome P450); (***ii***) biosynthesis of other secondary metabolites (i.e. neomycin, kanamycin and gentamicin biosynthesis, and penicillin and cephalosporin biosynthesis), and (***iii***) metabolism of terpenoids and polyketides (i.e. biosynthesis of enediyne antibiotics) (Supplementary Table S10). For *D. reticulatum*, the dominant predicted pathways were recorded for (***i***) xenobiotics biodegradation and metabolism (e.g. atrazine degradation, chloroalkane and chloroalkene degradation, aminobenzoate degradation, toluene degradation, xylene degradation) and (***ii***) metabolism of terpenoids and polyketides (e.g. biosynthesis of siderophore group nonribosomal peptides, biosynthesis of type II polyketide products, nonribosomal peptide structures, and type I polyketide structures) (Supplementary Table S10).

## Discussion

To the best of our knowledge, this is the first survey addressing the prokaryotic community composition, diversity, specificity, and putative functionality of the sympatric southwestern Atlantic sponges *Ap. caissara*, *Ax. corrugata,* and *D. reticulatum*.

In an attempt to overcome the difficulty encountered in the traditional taxonomy, DNA barcoding was implemented to assist in the identification^7^. Due to the gene organisation not being conserved at the poriferan mitogenomes and the presence of certain linear ones^25^, it was difficult to amplify the standard barcoding fragment for eukaryotic animals, the mitochondrial gene for the subunit I of the cytochrome c oxidase (cox-1). Different markers were tested: a downstream region (I3M11) of cox-1^26^, internal transcribed spacer (ITS)^27^, and a short LSU rRNA fragment^28^. Only recently^28^, the C2-D2 region of 28S rDNA was proven to be universally suitable for barcoding all sponge classes, providing high resolution and easy amplification. The ITS could also provide resolution in the case of *Aplysina* species^29^, whereas, cox-1 was unable to separate species within the family Aplysinidae^30^.The cob gene is evidently capable of differentiating sponge species^14^; therefore, the cob gene provided robust separation between *Ax. corrugata* and *D. reticulatum*, and it could separate *Ap. caissara* from all other *Aplysina* species. However, cob was unable to differentiate the other five *Aplysina* species and care should still be taken when barcoding the family Aplysinidae. There is only one cob sequence for *Ax. corrugata* (NC006894) available at the GenBank; however, it showed less than 83% sequence similarity to our sequence and was grouped outside the *Ax. corrugata* and *D. reticulatum* cluster. Hence, with a more comprehensive sampling, the cob gene may help improve the molecular identification of these sponge species; although, it is outside the scope of the present contribution.

To summarise the differences in the prokaryotic structure associated with each sponge species and those found in the environmental samples, the alpha diversity indices were compared. Recently, the surrounding sediment has more often been included in the analysis but not as frequently as seawater^10,31,32^. Sediment presented the highest values for richness, diversity, and evenness indices. Furthermore, sediment did not approach a plateau at the rarefaction curve, which was observed in other studies^10,31,32^. Surprisingly, seawater reached the plateau, even though it presented the second highest values for observed richness investigated in this survey. Independently of the high-throughput sequence platform used, seawater is typically asymptote^10,32–34^, whereas it might approach the plateau when a rarefied dataset is used^31^. It was observed that the prokaryotic richness in most of the sponges exhibited a less complex community than those found in seawater and sediment^10^. Similarly, *Ap. caissara*, *Ax. corrugata*, and *D. reticulatum* presented lower observed richness than the ones registered in sediment, whereas *Ap. caissara* and *D. reticulatum* may approach the richness registered for seawater. In contrast, for diversity and evenness indices, the sponge species had values in-between sediment and seawater. Cleary and colleagues^31^ detected a correlation between evenness and richness; biotypes with high evenness also showed the highest richness. In the present study, a shift was detected between *Ap. caissara* and *D. reticulatum*, where the former had higher richness and lower evenness compared to the latter. The evenness might have played an essential role by (***i***) regulating the community resistance to invasion, (***ii***) maintaining the functional stability of an ecosystem, and (***iii***) responding to the effect of an unexpected selective stress^35,36^. The high evenness registered from sponge species indicates that these species would be able to overcome invasion and stress while retaining the functionality of their communities.

In the present study, 40–47 distinct prokaryotic phyla were associated with three sponge species, which was within the range of 41–72 detected in sponges worldwide^10,12^. Regardless of the technique used, the most dominant prokaryotic phyla associated with sponges were affiliated to Proteobacteria, Chloroflexi, Cyanobacteriota, Acidobacteriota, and Actinobacteriota^10,31,32,37^. In the present survey, these were also the most dominant, along with Bacteria (unclassified) and Crenarchaeota. The most dominant prokaryotic communities associated with other *Aplysina* collected in seven sampling places (*A. aerophoba*, *A. cavernicola*, *A. fulva*, *A. archeri*, and *A. cauliformis*) were Proteobacteria (relative abundance of ~ 15%), unclassified (~ 12%), Chloroflexi (~ 15%), Acidobacteriota (~ 8%), and Actinobacteriota (~ 8%), which were distinct from the ones detected for *Ap. caissara* in the present study in terms of associated-phylum and relative abundances^10^. Likewise, for *Ax. corrugata* collected at the Bahamas and United States, the most dominant prokaryotic phyla were Proteobacteria (~ 15%), Crenarchaeota (~ 12%), unclassified (~ 5%), and Cyanobacteriota (~ 5%)^10^, which were again different from the ones associated with the Brazilian *Ax. corrugata*. Using a cloning library, seven phyla were detected associated with *D. reticulatum* collected from the north coast of São Paulo, in which the most dominant were Cyanobacteria and Proteobacteria^38^, contrasting with the results obtained in the present survey. Certain studies demonstrated that the most abundant classes associated with several sponges collected worldwide, including five *Aplysina* species and *Ax. corrugata*, were Gammaproteobacteria and Alphaproteobacteria^10,32^, whereas in the present survey, the most abundant were Bacteria (unclassified), Gammaproteobacteria, Alphaproteobacteria, Nitrososphaeria, and Cyanobacteriia. Furthermore, the prokaryotic communities associated with the three sponge species were markedly different from the ones obtained from seawater and sediment. Based on the differences in the relative abundance and community compositions associated with the sympatric species *Ap. caissara*, *Ax. corrugata*, and *D. reticulatum,* the host seemingly plays a deterministic role in shaping the structure of their own prokaryotic communities. These complex sponge-prokaryotic associations are a defined pattern with robust supportive evidence from numerous sponge species inhabiting the environmentally diverse marine habitats and climate zones^10,33,39,40^. The differences that encompassed three sympatric sponge species, of which two were phylogenetically related, might also be correlated to the evolutionary history between the symbionts and the sponge species. The unclassified OTUs associated with *Ap. caissara*, *Ax. corrugata,* and *D. reticulatum* further confirmed that the Brazilian sponge species represent a reservoir of novel prokaryotic diversity.

In addition to the assessment of the prokaryotic community composition and diversity, an attempt was made to correlate the capability of performance by the prokaryotic lineages enhanced in each sponge species, and their corresponding predicted functions registered. Notably, the functional prediction based on the 16S rRNA gene must be validated by metagenomics or whole genome sequencing. However, our approach was adequate, as it allowed the inclusion of prokaryotic genomes of sponges, seawater, and sediment to the dataset provided by the software, although a bias was observed within the taxonomic affiliation of the genomes. The majority of genomes in marine sponges were affiliated with Actinobacteria and Proteobacteria, which are well known to contain several secondary metabolites, whereas *Streptomyces* accounted for 30% of the bacteria isolated from marine sponge capable of producing antimicrobial compounds^41^. A significant effort has been made to sequence bacterial genomes that exhibit biotechnological potential, demonstrating a huge gap in the knowledge regarding the capability of the bacteria associated with marine sponges. Even with this bias, the approach provided insights into the potential functional aspects of the prokaryotic communities associated with three Brazilian sponge species. As described below, several correlations could be made between prokaryotic members and predicted functions, whereas many lineage(s) capable of performing them are yet to be discovered.

Among all the prokaryotic phyla and class levels in this survey, 54 were responsible for the variability detected among categories. The phyla enhanced in *Ap. caissara* have been associated with other marine sponges, including several *Aplysina* species, with relative abundances ranging from 0.001 to approximately 32%. Besides, some of these phyla contained several sponge-enriched clusters^9,42–44^. Metagenome-assembled genomes (MAGs) obtained from *Ircinia ramosa* demonstrated that Chloroflexi and Dadabacteria participated in nitrogen and sulphur cycling. In contrast, Nitrospinota were involved in sulphur cycling, and these three phyla were capable of synthesising distinct B-vitamins^45^. Furthermore, Chloroflexi associated with *Ap. aerophoba* showed features for glycolysis, carbon fixation, machinery for transcription, purine and pyrimidine metabolism, biosynthesis of most amino acids and cofactors, and potential aromatic degradation^46^, as well as halogenases involved in the production of brominated compounds^47^. Additionally, a Chloroflexi bacterium was the likely producer of a nonribosomal peptide synthase (NRPS)^48^. Several functional capabilities were detected in the genomes of marine Dadabacteria, Nitrospinota, and Deinococcota^49–54^, which were enhanced in *A. caissara*; however, further research is required to corroborate their performance in the sponge. Metabolic reconstruction of the metagenome of *Ap. aerophoba* and *Petrosia ficiformis* revealed that PAUC34f members contained genes (***i***) involved in glycolysis and oxidative phosphorylation, (***ii***) encoding numerous enzymes involved in the uptake and/or metabolism of nitrogen and sulphate and in the production of amino acids, vitamins, purines and pyrimidines, and (***iii***) encoding polyketides (PKS) modules and proteins, and several secondary metabolites biosynthesis gene clusters^55^. Several of the classes enriched in *Ap. caissara* have been associated with sponge species^46,56^. Among them, the best characterised is Dehalococcoidia, detected in numerous sponges including several *Aplysina* species, but with relative abundances of only 0.25%^46,56^. Marine Dehalococcoidia has been shown to be involved in the biogeochemical cycling of carbon, methane, and sulphur, and in bioremediation, especially of the chloroorganic pollutants. *Aplysina* species are well known for their halogenated substances, including the brominated compounds, which might be used by Dehalococcoidia. Hence, it may play an essential role in disarming the chemical defence systems and in attenuating signal agents by dehalogenating the halogenated signalling molecules^57–61^. To the best of our knowledge, genome and functional information are unavailable for members of the other classes enhanced in *Ap. caissara*; thus, no inference can be derived at present. Overall, the features described above were predicted in *Ap. caissara* and it is tempting to speculate that they might be performed by the lineages enriched in this sponge host. For instance, Chloroflexi, PAUC34f, and Deinococcota might be responsible for the metabolism of terpenoids and PKS and the biosynthesis of other secondary compounds, whereas metabolism of amino acid, cofactors, and vitamins might be performed by all phyla, except Dadabacteria. Additionally, it is tempting to speculate that Dehalococcoidia might be responsible for the energy metabolism and xenobiotics biodegradation predicted in *Ap. caissara*. Furthermore, as several members were involved in the biogeochemical cycling of nitrogen, sulphur, and carbon, it might indicate that functional redundancy is occurring in this host. To provide further support to the lineages that might be involved in the predicted functions enriched in *Ap. caissara* and their specificity, these taxonomic levels comprised 3202 OTUs detected in *Ap. caissara*, from which 2931 were host specific.

The class TK-10, enriched in *D. reticulatum*, was the producer of halogenases in several *Aplysina* species an essential enzyme in the biosynthesis of brominated and chlorinated secondary metabolites^58^. Dragmacidins are bromoindole alkaloid that inhibit the growth of several cancer cell lines and present antimicrobial and anti-inflammatory activities^62,63^. Thus, it can be considered that TK-10 are involved in the metabolism of terpenoids and PKS as predicted in *D. reticulatum*. The class Planctomycetes enriched in *D. reticulatum* was until recently classified as a phyla^64^. Planctomycetes are enriched with the capacity to synthesise secondary metabolites (e.g. PKS and NRPS genes, and antimicrobial and anticancer compounds) and gene clusters responsible for biofilm formation and quorum sensing^65,66^. Moreover, Planctomycetes are also able to degrade herbicides^66^. Thus, this phylum might be responsible for cellular community, metabolism of terpenoids and PKS, and degradation and metabolism of xenobiotics. Furthermore, the class Parcubacteria have small genomes and cell sizes, and important gaps in core metabolic potential, consistent with a symbiotic lifestyle, and they display relatively low abundance in surface seawater. The features detected in the genomes obtained from several places, including marine, were amino acids and fatty acid metabolism, membrane transport, secondary metabolites biosynthesis, and aromatic compound degradation^67–69^. These characteristics were predicted in *D. reticulatum;* thus, Parcubacteria might be involved in these activities. Analyses of certain marine Desulfobacterota genomes revealed that the major predicted clusters of orthologous groups were correlated to amino acid transport and metabolism, carbohydrate transport and metabolism, and lipid transport and metabolism^70,71^. These features predicted in *D. reticulatum* might be performed by Desulfobacterota, even though the unclassified members were enhanced. Nanoarchaeota have thus far been detected in extreme environments; they are obligate symbionts of other Archaea, consequently they contain reduced genomes, and lack most biosynthetic pathways and functional ATPase has not been detected^72–75^. Based on the abovementioned and on the fact that sponges were not collected from extreme habitats, and none of the predicted functions detected in *D. reticulatum* were observed in the genomes of Nanoarchaeota, seemingly this phylum is indeed symbiont of another Archaea that is associated with *D. reticulatum*. In this study, Nanoarchaeota was for the first time detected in marine sponges and the functional and ecological relationships among host and their associated microbiota are yet to be addressed. Furthermore, insufficient information was obtained for several lineages enhanced in *D. reticulatum*. These members comprised 1283 OTUs detected in *D. reticulatum*, from which 792 were host specific, further validating that the enriched members were likely to be performing the predicted functions.

The only member enriched in *Ax. corrugata* was Bacteria that could only be classified at the domain level; thus, no correlation could be drawn presently. It highlights that although our knowledge regarding the prokaryotic diversity has developed in the last decade, there is still more to be discovered. To further corroborate that this lineage is most likely carrying out the predicted functions, 1471 OTUs from a total of 1625 were host specific.

Overall, in the present study, it was demonstrated that the prokaryotic communities associated with *Ap. caissara*, *Ax. corrugata*, and *D. reticulatum* follow a deterministic-based assembly mechanism. The sponge species play a pivotal role in selecting their own prokaryotic communities, displaying host specificity. The unclassified OTUs associated with Brazilian sponge species represented an untapped reservoir of prokaryotic diversity corroborating the hotspot ecoregions hypothesis*.* Although 64 prokaryotic phyla comprising 169 classes were detected, only 54 lineages were responsible for the variability detected among categories. Surprisingly, the lineages associated with three sponge species could be correlated with the predicted functions enriched in each host. Although the predictive nature of the functional profiling based on 16S rRNA marker is not a substitute for the whole metagenome profiling in microbial ecology surveys, it provides a simple and cheap assessment of putative functions in the community.

## Material and methods

### Sponges and environmental sampling

Samples were collected at the southern rock shores of Guaecá-Prainha, São Sebastião (23° 49′ 22.8′' S, 45° 28′ 19.2′' W), São Paulo state, Brazil, southwestern Atlantic, in March 2019. Measurements of salinity and temperature at the time of sampling were 33.4 ppm and 29.4 °C, respectively. Five individuals of each species, *Aplysina caissara* (endemic) (Pinheiro & Hajdu, 2001) (Demospongiae, Verongiidiia, Aplysinidae), *Dragmacidon reticulatum* (Ridley & Dendy, 1886) (Demospongiae, Axinellida, Axinellidae) and *Axinella corrugata* (George & Wilson, 1919) (Demospongiae, Axinellida, Axinellidae) were collected by diving at depths of 5–6.5 m and placed separately in situ in sterile ziplock bags containing natural seawater. In situ images of the specimens were taken to aid in identification. Under similar conditions, five surrounding seawater samples (1 L each, approximately 1 m around the vicinity of the sponge specimens) were collected in 1 L sterile plastic bottles, along with sediment samples that were placed in sterile ziplock bags (approximately 2 kg each sample). Samples were placed in cooling boxes, transported to the laboratory (*c*. 30 min) at Center for Marine Biology of São Paulo University (CEBIMar/USP) for initial processing. Prior to the processing, the sponge specimens were rinsed with sterile artificial seawater^76^ to remove loosely associated organisms. Voucher samples were preserved in 70% ethanol for taxonomic identification. Pieces from the inner part of the sponge specimens were preserved in RNAlater (QIAGEN, Hilden, Germany) at 4 °C overnight and subsequently transferred to -20 °C, and environmental samples were maintained at -20 °C until further use.

### Sponge identification

Morphological identification of sponges was performed using standard methods: spicule and skeletal preparations followed Hajdu and colleagues^77^, and spongin fibres were prepared according to Pinheiro and Hajdu^3^. Phylogenetic inference (DNA barcoding^7^) was used in the molecular identification of species. Polymerase chain-reaction (PCR) amplifications were performed on sponge genomic DNA (see below) targeting the cytochrome b (cob) with primer pair Diplo-cob-f1m (5´-ATGTNTTNCCTTGRGGWCAAATGTC-3´)^78^ and Diplo-cob-r1 (5´-GGATTGAWCGTAAWATWGCRTAAGC-3´) modified from Lavrov and colleagues^78^. The reaction mixture (25 mL) contained 1.0 µL of template DNA (~ 20 ng), 2X buffer GoTaq (Promega, Madison, USA), 0.24 mM of each primer. The initial cycle of 4 min at 94 °C, 1 min at 47 °C and 1 min at 72 °C was followed by 35 cycles of 1 min at 92 °C, 1 min at 47 °C and 1 min at 72 °C for template amplification. A final extension of 6 min at 72 °C was used to complete the reaction. All PCR amplifications were carried out in a C1000 Touch thermal cycler (Bio-Rad, Hercules, CA, USA). Amplicons were visualised after electrophoresis on 1% agarose gel under UV light. The PCR products of the expected size were subjected to sequencing with the chain termination method in an ABI 3500 automatic sequencer (Applied Biosystems, Foster City, CA, USA) using the forward primer. The generated sequences were quality inspected and edited with the Sequence Scanner 2 software (Applied Biosystems, Foster City, CA, USA). To investigate the capacity of the cob to separate *Aplysina* species, voucher representatives obtained from Coleção de Porifera from the Universidade Federal de Pernambuco (UFPE) were subjected to the same procedures and analyses as described above. The resulting sequences were submitted to the NCBI database under the accession numbers MW092782-MW092802.

### Total community DNA extraction

Genomic DNA of internal sponge body (approximately 0.25 g) was extracted using DNeasy PowerSoil DNA isolation kit (QIAGEN, Hilden, Germany) according to the manufacturer’s protocol. Seawater samples (1 L) were filtered through nitrocellulose filters (0.2 μm pore-size) (Merck Millipore, Burlington, MA, USA) using a vacuum pump. The filters were cut into small pieces and directly used for DNA extraction with the DNeasy PowerSoil DNA isolation kit following the manufacturer’s protocol. Sediment samples (2 kg) were mixed, sieved, and an aliquot of 0.25 g was used for DNA extraction as explained above.

### Phylogenetic analyses

In total, 15 sequences, five from each sponge species, were used for the phylogenetic analyses. Closest relatives were searched using the megaBLAST and BLASTn algorithms of the NCBI^79–81^. The sequences obtained in the present study and those retrieved from NCBI were aligned with MEGA software v.10^82,83^. The FASTA format file was opened using SeaView software v. 4^84^ and saved as PHYLIP for jModeltest and Maximum Likelihood as well as nexus for Bayesian phylogenetic analyses. The selection of the best model for the inference was performed by jModeltest v. 2.1.6^85,86^ using CIPRES Science Gateway website v. 3.3^87^; the general-time reversible model (GTR^88^) was selected with a discrete gamma distribution of among-site rate variation (Γ_4_) and a proportion of invariant sites (I). The optimal and bootstrap maximum-likelihood inferences were performed at CIPRES Science Gateway website v. 3.3^80^. An optimal maximum-likelihood tree was determined using RAxML 8.2.12^89^, with 1000 replicates, each starting from a random tree, with the GTR + I + G model. Maximum-likelihood bootstrap support was ascertained with the same software and model, using 1000 replicates. A Bayesian Markov chain Monte Carlo (MCMC) analysis was also conducted using MrBayes 3.2.6^90,91^, with two runs using four chains (Metropolis-coupling) with 1000 generations, and GTR + I + G model. All other options, including priors, were default values. The ‘burn-in’ period before the MCMC reached stationarity was defined as 25% of the initial trees. Tree sets from the posterior distribution of the two independent runs were concatenated from the sample of trees and assumed to be randomly sampled from the posterior probability distribution and 50% majority-rule consensus tree.

### 16S rDNA Illumina sequencing

An aliquot of the purified genomic DNA was submitted to the Functional Genomics Center of the Luiz de Queiroz College of Agriculture (ESALQ-USP) to perform 16S rRNA gene sequencing. Briefly, the V4-region of the 16S rRNA gene, targeting the prokaryotic community, was amplified with the primer pair 515F^92^ and 806R^93^. The reaction mixture (25 μL) encompassed of 2.5 µL of template DNA (20 ng μl^-1^), 0.20 mM of each primer, 2X PCRBio Ultra Mix (PCRBiosystems, Wayne, USA). The thermal cycle started with 3 min at 95 °C, followed by 30 cycles of 30 s at 95 °C, 30 s at 60 °C and 30 s at 72 °C. A final extension of 10 min at 72 °C was applied for reaction completion. The amplicons were subjected to Illumina sequencing using MiSeq platform.

### Analyses of Sequencing data

Illumina sequences were processed using Mothur v. 1.44^94^. A pipeline was optimised and executed. Briefly, paired raw reads were subjected to pre-processing through quality checking. Then, the dataset was reduced to non-identical sequences to decrease computational effort. Sequences were aligned using the reference SILVA seed v. 138 database (mothur-formatted), provided by Mothur^95,96^. The dataset was reduced to non-redundant sequences and were pre-clustered. Then, chimeric sequences detected with UCHIME^97^ were removed from the dataset. The remaining sequences were phylogenetically classified. Undesirables and singletons were removed from the dataset. Sequences were assigned to OTUs classified at 97% sequence similarity. The libraries were normalised. OTUs were further classified based on the SILVA non-redundant v. 138 database (mothur-formatted)^95,96^. All 16S rRNA datasets generated in this study were deposited as Sequence Read Archive in NCBI database with Bioproject ID: PRJNA665805 (SAMN1626881-SAMN16268843). For a detailed description of the pipeline used see Supplementary Material.

### Calculation of community metrics

Richness (Sobs, CHAO, and ACE), diversity (Shannon and inverse Simpson) and evenness (Pielou’s evenness) indicators were calculated using the R package vegan v. 2.5–6^98^. ANOVA was performed using R package vegan 2.5–6^98^ to test the significant difference in the obtained mean values from each index. For ANOVA, *p* value of < 0.001 was considered statistically significant. The multcomp version 1.4–13 R package^99^ was applied for multiple comparisons of mean values with Tukey contrasts. Statistical analyses were performed for the barchart when the sum of the relative abundance from all replicates for phyla and class were ≥ 5% and ≥ 1%, respectively, and were also carried out in R^100^. Square root transformation was used to improve the normality of the relative abundance. The Welch´s test was performed, followed by a Pos-Hoc test with Holm pairwise correction because of unequal homogeneity of variance.

The Venn diagram was constructed to determine the number of OTUs specifically assigned to each category and common to all categories using the R package VennDiagram v. 1.6.20^100,101^, whereas the identity of OTUs was discovered using the online tool available at http://bioinformatics.psb.ugent.be/webtools/Venn/. In this study, the core OTUs were defined as OTUs present in all categories but not necessarily in all replicates.

The unclassified OTUs associated with marine sponges at certain level of the taxonomic affiliation (from phylum to genus) were further investigated. These OTUs were subjected to BLASTn^79–81^ using two distinct databases: (***i***) type strain from NCBI (available on 02/01/2021) and (***ii***) SILVA non-redundant v. 138. It is important to note that standard NCBI annotation was used to collect host and isolation source information when available. The scripts used are provided in the Supplementary Material.

To determine the phylogenetic lineages responsible for the differences detected in each category (*i.e.* sponge species, seawater, and sediment), LEfSe v 1.0^102^ was performed on the Galaxy web platform^103^ with default parameters. Distances of the samples in each category and their respective group centroids were calculated based on Bray–Curtis distances using the function vegdist from the vegan package v. 2.5–6 in R^98,100^. nMDS using the Bray–Curtis dissimilarities was calculated in vegan package v. 2.5–6 in R^98,100^. The differences in Bray–Curtis dissimilarity among categories was tested using ADONIS.

### Functional predictions

To obtain insights regarding putative functions using the 16S rRNA gene, the software Tax4fun2 v. 1.1.5 was used^104^. The advantage of this programme is that in addition to the reference genome database provided, genomes of interest can also be included. To this end, genomes from marine sponges, seawater, and sediment from all five oceans were searched at NCBI^79,80^ using genomic DNA/RNA and RefSeq datasets with the following keywords: marine sponge, seawater, or sediment combined with bacteria or archaea. Nearly complete genome sequences with at least one copy of the 16S rRNA gene were further used. Thus, 123 prokaryotic genomes isolated from sponges and 241 and 720 genomes obtained from sediment and seawater, respectively, were analysed. The details, including taxonomic affiliations, of the added genomes can be found in Supplementary Table S1a and the pipeline used is described in Supplemental Material. Two tables were generated (functional and pathway predictions). The relative abundance of KO was analysed with nMDS in vegan package v. 2.5–6 in R^98,100^. LEfSe v 1.0 was also used here to identify the KEGG pathways as significant biomarkers for each category by calculating LDA^10251^, as explained above. Nonetheless, the results obtained with the predictive functional profiling using 16S rRNA marker gene does not substitute metagenomic profiling and functional gene annotation and may diverge. Furthermore, due to the functional overlap, some KOs were assigned to more than one pathway.

## Permits

Sampling was performed under the scientific collection permits A097B99 issued by Sistema Nacional de Gestão do Patrimônio Genético e do Conhecimento Tradicional Associado, 61460–2 issued by Sistema de Autorização e Informação sobre Biodiversidade do Instituto Chico Mendes de Conservação da Biodiversidade, both from Ministry of the Environment and 260108–001.161/2013 issued by Instituto Florestal, Secretaria do Meio Ambiente do Estado de São Paulo.

## Supplementary Information

Supplementary file

Supplementary figure 1.

Supplementary Table S1.

Supplementary Table S2.

Supplementary Table S3.

Supplementary Table S4.

Supplementary Table S5.

Supplementary Table S6.

Supplementary Table S7.

Supplementary Table S8.

Supplementary Table S9.

Supplementary Table S10.

Supplementary Legends.

## Data Availability

All 16S rRNA datasets generated through this study were deposited as Sequence Read Archive in NCBI database with Bioproject ID: PRJNA665805 (SAMN1626881-SAMN16268843). All cob sequences were deposited at NCBI database under Accession Numbers: MW092782-MW092802.
