# Supplementary material for: Community composition and functional prediction of prokaryotes associated with sympatric sponge species of southwestern Atlantic coast
Source: Sci Rep. 2021 May 5;11:9576. doi: 10.1038/s41598-021-88288-3 (PMC8100286; doi:10.1038/s41598-021-88288-3)
Supplement: Supplementary file 1 — Supplementary file [file 41598_2021_88288_MOESM1_ESM.docx]

**Prokaryotic community composition and functional prediction associated with sympatric sponge species from southwestern Atlantic coast**

Hardoim, C.C.P.^1*^; Ramaglia, A.C.M^1^; Lôbo-Hajdu, G.^2^; Custódio M.R.^3^

***Corresponding author.** E-mail cristianehardoim@gmail.com

**Supplementary Material**

***16S rDNA Illumina sequencing and analyses***

An aliquot of the purified genomic DNA was submitted to the Functional Genomics Center of the Luiz de Queiroz College of Agriculture (ESALQ-USP) for sequencing the 16S rRNA gene. Briefly, the V4-region of the 16S rRNA gene, targeting the prokaryotic communities, was amplified with the primer pair 515R (5´-GTGYCAGCMGCCGCGGTAA-3´)^1^ and 806R (5´-GGACTACNVGGGTWTCTAAT-3´)^2^. The reaction mixture (25 μL) encompassed of 2.5 uL of template DNA (20 ng/μl), 0.20 mM of each primer, 2X PCRBio Ultra Mix (PCRBiosystems, Wayne, USA). The thermal cycle started with 3 min at 95 °C, followed by 30 cycles of 30 sec at 95 °C, 30 sec at 60 °C and 30 sec at 72 °C. A final extension of 10 min at 72 °C was applied to complete the reaction. The amplicons were subjected to Illumina sequencing with MiSeq platform.

Illumina sequences were processed in Mothur v. 1.44^3^. A pipeline was optimized and executed. Thus, initially, the command make.contigs was used to join the paired raw sequences. Then, the demultiplexed fastq sequences had the first quality control using the command screen.seqs with parameters: fasta=, group=, maxambig=0, maxhomop=8, minlength=270, maxlength=320. The commands unique.seqs and count.seqs were used to reduce the dataset to non-identical sequences and thus minimize computational effort. Prior to the alignment of the non-redundant sequences, the positions at which the primer pair aligned in the 16S rRNA gene was searched using a sequence of *Escherichia coli* (J01851) and the reference SILVA seed v. 138 database (mothur-formatted), provided by Mothur^4^ with the command align.seqs and parameters fasta=ecoli.fasta, reference=silva.seed_v138.align. The positions that the reference database should be trimmed were shown by the command summary.seqs. Then the command pcr.seqs was carried out with parameters fasta=silva.seed_v138.align, start=11895, end=25318, keepdots=F and then aligned with align.seqs command and parameters fasta=, reference=silva.seed_v138.pcr.align. The sequences aligned to the expected position were maintained (screen.seqs: fasta=, count=, start=2, end=13423, criteria=90 and filtered with filter.seqs: fasta=, vertical=T, trump=.). Aligned sequences were reduced to non-redundant sequences with the command unique.seqs. Prior to chimera check, the sequences were pre-clustered (pre.cluster: fasta=, count=, diffs=2), which allowed up to two differences between sequences. Then, chimeric sequences were detected with UCHIME^5^ (chimera.uchime command with parameters: fasta=, count=, dereplicate=t) and filtered out with the command remove.seqs. The command pcr.seqs was applied again to trim the aligned SILVA non-redundant v. 138 database^4,6^ with the parameters fasta=silva.nr_v138.align, start=11895, end=25318, keepdots=F. Then, sequences were phylogenetically classified with the command classify.seqs with the parameters fasta=, count=, taxonomy=silva.nr_v138.tax, reference=silva.nr_v138.pcr.align, cutoff=80. The sequences classified as mitochondria, chloroplast, eukaryota, and unknown were filtered out with the command remove.lineage. Then, the commands cluster.split with parameters: fasta=, count=, taxonomy=, taxlevel=4, splitmethod=classify, cutoff=0.03, method=average, cluster=f were applied. This script also calculated the pairwise distances between aligned sequences (dist.seqs: fasta=, cutoff=0.03). The output was used in cluster.split(file=). To filter the list file generated in the previous script, the command remove.rare(list=, count=, nseqs=1) was used, which removed singletons from the data set. To list the sequences in the filtered file the command list.seqs(list=) was applied, followed by get.seqs(fasta=, taxonomy=, count=, accnos=) selecting filtered sequences from all files. Then, the command make.shared(list=, count=, label=0.03), where the shared file contained the operational taxonomic units (OTUs) classified at 97% sequence similarity. The libraries were normalized with the command sub.sample(shared=). In order to get the representative sequences from each OTU, the command get.oturep(list=, fasta=, count=, method=abundance) was applied. OTUs were further classified based on the SILVA non-redundant v. 138 database (mothur-formated)^4,6^ with the commands summary.tax(taxonomy=, count=) and classify.otu(list=, count=, taxonomy=, label=0.03). The rarefaction curve was calculated within Mothur with the command rarefaction.single and parameters shared=, freq=1000. All 16S rRNA data sets generated in this study were deposited as Sequence Read Archive in NCBI database with Bioproject ID: PRJNA665805 (SAMN1626881-SAMN16268843).

***Functional Predictions***

To get insights regarding putative function using the 16S rRNA gene the software Tax4fun2 v. 1.1.5 was used^7^. The advantage of this program is that in addition to the reference genome database provided, genomes of interest can also be included. To this end, genomes from marine sponges, seawater, and sediment from all five oceans were searched. It was done at NCBI^8,9^ using genomic DNA/RNA and RefSeq datasets with the following keywords: marine sponge, seawater, or sediment combined with bacteria or archaea. Nearly complete genome sequences contained at least one copy of the 16S rRNA gene were further used. Thus, 123, 241, and 720 prokaryotic genomes isolated from sponges, and obtained from sediment and seawater, respectively, were included in this analysis. The details, including taxonomic affiliations, of the added genomes can be found in Supplementary Table S1a and the pipeline used is described in Supplemental Material. The extraction of the 16S rRNA gene sequences from the genomes of interest was performed with extractSSU(genome_folder = "proka", file_extension = "fasta", path_to_reference_data = "Tax4Fun2_ReferenceData_V2"). A BLAST search using the SILVA SSURef database v. 132 was used to identify the 16S rRNA gene sequences^4,9^. The prokaryotic genomes were assigned functional annotations with assignFunction(genome_folder = "proka/", file_extension = "fasta", path_to_reference_data = "Tax4Fun2_ReferenceData_v2", num_of_threads = 14, fast = T, path_to_diamond_binary_mac = "diamond"). BLASTp with diamond was used to create the functional profiles against the KEGG orthology (KO, *i.e.*, set of homologous sequences) database^10^. Prior to functional annotation, protein sequences were predicted using prodigal v. 2.6.3^11^. The output from these two scripts were used to build the user-defined database with generateUserDataByClustering(path_to_reference_data = "Tax4Fun2_ReferenceData_v2", path_to_user_data = "proka", name_of_user_data = "Proka_Ref1", SSU_file_extension = "_16SrRNA.ffn", KEGG_file_extension = "_funPro.txt", use_force = T) using uclust^12^. The first step in the functional assignments was to run BLAST including the user reference data [runRefBlast(path_to_otus = "proka_rep_otus.fasta", path_to_reference_data = "Tax4Fun2_ReferenceData_v2", path_to_temp_folder = "Proka_Ref99NR", database_mode = "Ref99NR", use_force = T, num_threads = 14, include_user_data = T, path_to_user_data = "proka", name_of_user_data = "Proka_Ref1")], followed by the functional assignments [makeFunctionalPrediction(path_to_otu_table = "proka_otu_table.txt", path_to_reference_data = "Tax4Fun2_ReferenceData_v2", path_to_temp_folder = "Proka_Ref99NR", database_mode = "Ref99NR", min_identity_to_reference = 0.97, normalize_pathways = F, normalize_by_copy_number = T, include_user_data = T, path_to_user_data = "proka", name_of_user_data = "Proka_Ref1")]. Two tables were generated (functional and pathway predictions). The relative abundance of KOs was analyzed with nMDS in vegan package v. 2.5-6 in R^13,14^. LEfSe v 1.0 was also used here to identify the KEGG pathways as significant biomarkers for each category by calculating the linear discriminant analysis (LDA)^15^, as explained in the main text. Nonetheless, the results obtained with the predictive functional profiling using 16S rRNA marker genes do not substitute metagenomic profiling and functional gene annotation and they can diverge. Furthermore, due to the functional overlap, some KOs could be assigned to more than one pathway.

***Scripts for Blast analyses***

*# Database from SILVA_138 release*

makeblastdb -in SILVA_138.1_SSURef_NR99_tax_silva.fasta -out Silva_138 -dbtype nucl -title ‘SILVA 16S rRNA Ref NR_99 rel_138’ -parse_seqids

*#Run blast against SILVA database*

blastn -query unclassified.fasta -db /home/dell-ubuntu/Documents/Databases/Silva_138 -max_target_seqs 1 -evalue 1e-20 -outfmt '6 qseqid sseqid pident evalue' -out unclassified_Silva.txt -num_threads 4

*#Run blast against NCBI Type Strain database*

blastn -query unclassified.fasta -db /home/dell-ubuntu/Documents/Databases/16S_ribosomal_RNA -max_target_seqs 1 -evalue 1e-20 -outfmt '6 qseqid sseqid pident evalue' -out unclassified_16S_TS.txt -num_threads 4

**List of references:**

1. Parada, A.E., Needham, D.M. & Fuhrman, J.A. Every base matters: Assessing small subunit rRNA primers for marine microbiomes with mock communities, time series and global field samples. *Environ. Microbiol.* **18**, 1403–1414 (2016).

2. Apprill, A., Mcnally, S., Parsons, R. & Weber, L. Minor revision to V4 region SSU rRNA 806R gene primer greatly increases detection of SAR11 bacterioplankton. *Aquat. Microb. Ecol.* **75**, 129–137 (2015).

3. Schloss, P.D. *et al.* Introducing mothur: Open-source, platform-independent, community-supported software for describing and comparing microbial communities. *Appl. Environ. Microbiol.* **75**, 7537–7541 (2009).

4. Quast, C. *et al.* The SILVA ribosomal RNA gene database project: Improved data processing and web-based tools. *Nucleic Acids Res.* **41**, (2013).

5. Edgar, R.C., Haas, B.J., Clemente, J.C., Quince, C. & Knight, R. UCHIME improves sensitivity and speed of chimera detection. *Bioinformatics* **27**, 2194–2200 (2011).

6. Yilmaz, P. *et al.* The SILVA and ‘all-species Living Tree Project (LTP)’ taxonomic frameworks. *Nucleic Acids Res.* **42**, 643–648 (2014).

7. Wemheuer, F. *et al.* Tax4Fun2: prediction of habitat-specific functional profiles and functional redundancy based on 16S rRNA gene sequences. *Environ. Microbiome* **15**, 11 (2020).

8. Agarwala, R. *et al.* Database resources of the National Center for Biotechnology Information. *Nucleic Acids Res.* **44**, D7–D19 (2016).

9. Altschul, S.F., Gish, W., Miller, W., Myers, E. W. & Lipman, D. J. Basic Local Alignment Search Tool 2Department of Computer Science. *J. Mol. Biol.* **215**, 403–410 (1990).

10. Buchfink, B., Xie, C. & Huson, D.H. Fast and sensitive protein alignment using DIAMOND. *Nat. Methods* **12**, 59–60 (2014).

11. Hyatt, D. *et al.* Prodigal: Prokaryotic gene recognition and translation initiation site identification. *BMC Bioinformatics* **11**, (2010).

12. Edgar, R.C. Search and clustering orders of magnitude faster than BLAST. *Bioinformatics* **26**, 2460–2461 (2010).

13. Oksanen, A.J. *et al.* vegan: Community Ecology Package. R package version 2.5-6. https://CRAN.R-project.org/package=vegan (2019).

14. R Core Team. R: A language and environment for statistical computing. R Foundation for Statistical Computing, Vienna, Austria. URL <https://www.R-project.org/>. ( 2020).

15. Segata, N. *et al.* Metagenomic biomarker discovery and explanation. *Genome Biol.* **12**, (2011).
