# Supplementary material for: Community composition and functional prediction of prokaryotes associated with sympatric sponge species of southwestern Atlantic coast
Source: Sci Rep. 2021 May 5;11:9576. doi: 10.1038/s41598-021-88288-3 (PMC8100286; doi:10.1038/s41598-021-88288-3)
Supplement: Supplementary file 13 — Supplementary Legends. [file 41598_2021_88288_MOESM13_ESM.docx]

**Legends to Supplementary Figure and Tables**

**Figure S1: Richness for each category.** *Ap. caissara* (Ac), *Ax. corrugata* (Ax), *D. reticulatum* (Dr), seawater (SW), and sediment (SD) with normalized libraries and replicates from each category pooled (n = 5).

**Table S1**: **Functional prediction.** Details of the genomes included in the analyses (a), Taxonomic affiliation of the genomes associated with marine sponges (b), obtained from seawater (c) and sediment (d).

**Table S2: OTU *vs*. samples table.** All categories and their respectively OTU and taxonomic assignments.

**Table S3:** **Statistical analyses for the alpha diversity indexes.** Ac: *Aplysina caissara*, Ax: *Axinella corrugata*, Dr: *Dragmacidon reticulatum*, SW: seawater, SD: sediment, Sobs: estimated richness.

**Table S4:** **Prokaryotic community composition.** Taxonomy composition detected in (a) all categories, (b) *Aplysina caissara*, (c) *Axinella corrugata*, (d) *Dragmacidon reticulatum*, (e) seawater, and (f) sediment

**Table S5: Relative abundance of community composition.** Relative abundance at the (a) phylum and (b) class levels.

**Table S6. Blast of the unclassified OTUs associated with sponge species.** Closest match to the unclassified OTUs using NCBI Type Strain database (a), Phylogenetic distribution of the unclassified OTUs using NCBI Type Strain database (b), Closest match to the unclassified OTUs using Silva database (c), and Phylogenetic distribution of the unclassified OTUs using Silva database (d). *number of sequences

**Table S7: Venn diagram shared and exclusive OTUs**. Taxonomic composition of OTUs obtained exclusively in (a) *Aplysina caissara*, (b) *Axinella corrugata*, (c) *Dragmacidon reticulatum*, (d) seawater, (e) sediment, and the core (f)

**Table S8:** **LEfSe features**. List of features provided by LEfSe that are differential among sponge species, seawater and sediment with statistical and biological significance and ranked according to the effect size (LDA).

**Table S9:** **Relative abundance of the KEGG Orthologs (KO).** KOs relative abundance across the categories (sponge, seawater and sediment). Ac = *Aplysina caissara*, Ax = *Axinella corrugata*, Dr = *Dragmacidon reticulatum* SW = seawater, SD = sediment.

**Table S10:** **KEGG pathways information**. Full list of KEGGs for each sponge species with the pathway identifier, functional, pathway name and pathway class, LDA and corresponding *p*-value
